# Protective Role of Shiitake Mushroom-Derived Exosome-Like Nanoparticles in D-Galactosamine and Lipopolysaccharide-Induced Acute Liver Injury in Mice

**DOI:** 10.3390/nu12020477

**Published:** 2020-02-13

**Authors:** Baolong Liu, Yizhu Lu, Xingyi Chen, Philma Glora Muthuraj, Xingzhi Li, Mahesh Pattabiraman, Janos Zempleni, Stephen D. Kachman, Sathish Kumar Natarajan, Jiujiu Yu

**Affiliations:** 1 Department of Nutrition and Health Sciences, University of Nebraska-Lincoln, Lincoln, NE 68583, USA; baolong@huskers.unl.edu (B.L.); lyzgjyz6@hotmail.com (Y.L.); xychen@huskers.unl.edu (X.C.);; 2 Department of Chemistry, University of Nebraska-Kearney, Kearney, NE 68849, USA; pattabiramm2@unk.edu; 3 Department of Statistics, University of Nebraska-Lincoln, Lincoln, NE 68583, USA

**Keywords:** shiitake mushrooms, exosomes, nanoparticles, NLRP3 inflammasome, inflammation, fulminant hepatic failure

## Abstract

Fulminant hepatic failure (FHF) is a rare, life-threatening liver disease with a poor prognosis. Administration of D-galactosamine (GalN) and lipopolysaccharide (LPS) triggers acute liver injury in mice, simulating many clinical features of FHF in humans; therefore, this disease model is often used to investigate potential therapeutic interventions to treat FHF. Recently, suppression of the nucleotide-binding domain and leucine-rich repeat related (NLR) family, pyrin domain containing 3 (NLRP3) inflammasome, was shown to alleviate the severity of GalN/LPS-induced liver damage in mice. Therefore, the goal of this study was to find dietary exosome-like nanoparticles (ELNs) with therapeutic potential in curbing FHF by suppressing the NLRP3 inflammasome. Seven commonly consumed mushrooms were used to extract ELNs. These mushrooms were found to contain ELNs composed of RNAs, proteins, and lipids. Among these mushroom-derived ELNs, only shiitake mushroom-derived ELNs (S-ELNs) substantially inhibited NLRP3 inflammasome activation by preventing inflammasome formation in primary macrophages. S-ELNs also suppressed the secretion of interleukin (IL)-6, as well as both protein and mRNA levels of the *Il1b* gene. Remarkably, pre-treatment with S-ELNs protected mice from GalN/LPS-induced acute liver injury. Therefore, S-ELNs, identified as potent new inhibitors of the NLRP3 inflammasome, represent a promising class of agents with the potential to combat FHF.

## 1. Introduction

Fulminant hepatic failure (FHF) is a rare liver disease characterized by abrupt and extensive death of hepatocytes, as well as inflammation, coagulopathy, and hepatic encephalopathy [1,2]. The mortality of FHF in most cases is approximately 40%–80% [2,3]. Liver transplantation has been the most effective therapeutic modality for treating FHF in clinical practice, but the availability of donor livers is often limited [1,2]. Therefore, identifying new agents that have therapeutic potential in treating FHF is highly desirable.

Administration of D-galactosamine (GalN) and a low dose of lipopolysaccharide (LPS) triggers acute liver damage in mice, simulating many of the clinical manifestations of FHF in humans [4]. Therefore, GalN/LPS-induced acute liver injury in mice is used extensively to investigate the molecular mechanisms and potential therapeutic interventions of FHF [5,6,7]. The nucleotide-binding domain and leucine-rich repeat related (NLR) family, pyrin domain containing 3 (NLRP3) inflammasome has been found to play a critical role in GalN/LPS-induced acute liver injury in mice. mRNA and protein levels of the *Nlrp3* gene are increased in mice after GalN/LPS challenge [8,9], and inhibition of the NLRP3 inflammasome with its specific inhibitor MCC950 ameliorates the severity of GalN/LPS-induced acute liver injury in mice [6].

The NLRP3 inflammasome is a multimeric protein complex that contains NLRP3, apoptotic speck protein containing a caspase recruitment domain (ASC), and Caspase 1 (Casp1) [10,11]. NLRP3 inflammasome activation requires both priming and activating signals [12,13]. LPS, a component of the outer membrane of Gram-negative bacteria, often serves as a priming signal that induces transcription of the *Nlrp3* and *Il1b* genes and primes NLRP3 protein via post-translational modifications. A variety of molecules, including free fatty acid (FFA), bacterial toxin, extracellular ATP, or cholesterol crystals, could serve as activation signals to promote the assembly of the inflammasome protein complex. Upon formation of the NLRP3 inflammasome, Casp1 is activated and autocleaves itself to generate Casp1 p10 and p20. Casp1 also cleaves (1) pro-interleukin (IL)-1β and pro-IL-18 to generate the mature cytokines IL-1β and IL-18 and (2) Gasdermin D to trigger pyroptotic cell death [14,15].

The goal of the current study was to identify food-derived components that have therapeutic potential in treating FHF by suppressing the activity of the NLRP3 inflammasome. Dietary exosomes or exosome-like nanoparticles (ELNs) have emerged as a new class of agents with high translational potential [16,17,18,19]. Dietary exosomes or ELNs are nanoparticles that contain biomolecules, including lipids, RNAs, and proteins [18,20,21]. The nanoparticles from bovine milk have been authenticated as exosomes through verification via immunoblot analysis of exosome-specific surface markers on these vesicles [18,22]. However, because exosome-specific surface markers of nanoparticles from vegetables and fruits have not been established, these nanoparticles are called exosome-like nanoparticles, or ELNs. Food-derived exosomes and ELNs have been reported to regulate the functions of a variety of cells in mice and humans, and have beneficial effects on consumer health [18,19,23,24]. However, it was not clear whether any edible mushrooms contained ELNs and whether any ELNs with anti-inflammasome activity played a protective role in GalN/LPS-triggered acute liver injury in mice.

## 2. Materials and Methods

### 2.1. Isolation and Characterization of ELNs

The mushrooms were subjected to ELN isolation and characterization, as previously reported [20,21,25]. Seven fresh mushrooms, including white beech (*hypsizygus tessellatus*), brown beech (*hypsizygus tessellatus*), white button (*agaricus bisporus*), Swiss brown (*agaricus bisporus*), king oyster (*pleurotus eryngii*), shiitake (*lentinula edodes*), and oyster (*pleurotus ostreatus*), were obtained from local grocery stores. For each type, a whole mushroom (cap and stem) was minced, and 5–10 g of minced mushroom were ground for 15 s in cold phosphate-buffered saline (PBS) in a kitchen blender. The mushroom juice was subjected to sequential centrifugation at 500× *g* for 10 min, 2000× *g* for 20 min, 10,000× *g* for 30 min, and 100,000× *g* for 2 h. The ELN pellet was rinsed with PBS, resuspended in PBS or culture medium, and filtered using a 200 nm Acrodisc filter (Pall Laboratory, Port Washington, NY, USA). A NanoSight NS300 instrument (Malvern, Westborough, MA, USA) was used to measure the yield and size of ELNs. Images of ELNs were taken using scanning electron microscopy (SEM) as described [20]. RNAs from ELNs were purified using a miRNeasy Mini kit (Qiagen, Germantown, MD, USA) and separated on an agarose gel. Proteins were extracted from ELNs using lysis buffer containing 150 mM NaCl, 0.5% NP-40, 50 mM Tris-HCl (pH7.5), run on a Bis-Tris protein gel, and visualized with Coomassie blue staining. Lipids of ELNs were purified using the Folch method [24,26], loaded on a Silica gel thin-layer chromatography (TLC) plate (EMD Millipore, Burlington, MA, USA), separated using a chloroform/methanol/acetic acid mixture (190:9:1, Sigma, St. Louis, MO, USA), and stained with 10% CuSO_4_ in 8% phosphoric acid solution (Sigma).

### 2.2. Macrophage Cell Culture

Bone marrow-derived macrophages (BMDMs) were prepared as previously reported [20,27,28]. Briefly, the bone marrow cells from femur and tibia bones of C57BL/6J mice were collected and cultured in Roswell Park Memorial Institute (RPMI) 1640 medium (Corning, Tewksbury, MA, USA) containing 10% fetal bovine serum (FBS) (Atlanta Biologicals, Minneapolis, MN, USA, S1150), 20% L929 cell-conditioned medium, 50 μg/mL PenStrep (Corning), 2 mM glutamine (Corning), 1 mM sodium pyruvate (Corning), and 10 mM HEPES buffer (Corning). Cells were cultured at 37 °C in 5% CO_2_ for 6–8 days until macrophages reached 80%–90% confluence. To assess the anti-NLRP3 inflammasome activity of mushroom-derived ELNs, BMDMs were incubated with these ELNs for 16 h, treated with the priming signal LPS (InvivoGen, San Diego, CA, USA, tlrl-peklps, 10 ng/mL) for 3 h, then incubated with the activating signal, which included FFA sodium palmitate (Sigma, 1 mM, 16 h), alum (Thermo Scientific, Waltham, MA, USA, 0.5% *v*/*v*, 5 h), nigericin (Enzo Life Sci, Farmingdale, NY, USA, 5 μM, 30 min), and ATP (Sigma, 5 mM, 30 min). For activation of the Absent in Melanoma 2 (AIM2) inflammasome, BMDMs were treated with LPS for 3h followed by transfection of DNA (Sigma, D3664, 2 μg/well) for 2 h.

### 2.3. Mice

C57BL/6J mice from Jackson Laboratory (Bar Harbor, ME, USA) were maintained as described [20]. Animal experiments were conducted under the protocol (ID 1421) approved by the Institutional Animal Care and Use Committee of the University of Nebraska-Lincoln. To evaluate the effects of S-ELNs on acute liver injury induced by GalN/LPS, C57BL/6J mice were intraperitoneally injected with S-ELNs at the dose of 1 × 10^10^/g. The dose of S-ELNs was determined based on pilot pharmacokinetic tests and other groups’ studies [29,30,31]. Then, 48 h later, mice were intraperitoneally injected with a mixed solution of GalN (Sigma, 34539, 500 mg/kg) and LPS (Sigma, L2630, 15 µg/kg) to trigger acute liver damage. After 6 h, all mice were sacrificed, and serum and livers were taken for further analysis. One small piece of liver was fixed immediately in formalin solution (neutral buffered, 10%, VWR, Radnor, PA, USA), embedded in paraffin, and cut into 8-µm slices, which were placed on slides and subjected to routine Haemotoxylin and Eosin (H&E) staining or terminal deoxynucleotidyl transferase dUTP nick end labeling (TUNEL) staining. The levels of alanine aminotransferase (ALT) and aspartate aminotransferase (AST) in the serum were measured using a Vitros-250 Chemistry Analyzer (Johnson & Johnson, New Brunswick, NJ, USA).

### 2.4. Immunoblot Analysis, mRNA Extraction, and Quantitative PCR (qPCR)

Immunoblot analysis was conducted as described [20]. Primary antibodies used were: Anti-IL-1β goat antibody (R&D systems, Minneapolis, MN, USA, AF401NA, 1:2000); anti-tubulin rabbit polyantibody (Santa Cruz Biotechnology, Dallas, TX, USA, SC-5286, 1:200); anti-Casp1 (p10) mouse antibody (Adipogen, San Diego, CA, USA, AG20B0044C100, 1:1000); anti-ASC rabbit antibody (Adipogen, AG25B0006C100, 1:1000); anti-NLRP3 mouse antibody (Adipogen, AG20B0014C100, 1:1000); and anti-Nek7 rabbit antibody (Abcam, Cambridge, MA, USA, ab133514, 1:10000). RNA-bee (Tel-Test, Friendswood, TX, USA) was used to extract mRNAs from livers, which were then subjected to qPCR analysis [20]. The housekeeping genes, including the *Hprt* and *Rplp0* genes, were used as reference genes for normalization.

### 2.5. Lactate Dehydrogenase (LDH) Release Assay, Enzyme-Linked Immunosorbent Assay (ELISA), and ASC Speck Staining

All these assays were carried out as described [20]. The ELISA kits used included tumor necrosis factor α (TNFα) (BioLegend, San Diego, CA, USA, 430901); IL-6 (BioLegend, 431301); IL-18 (MBL, Worburn, MA, USA, D042-3); and IL-1β (eBioscience, San Diego, CA, USA, 88701388). CytoTox 96 Nonradioactive Cytotoxicity Assay kit (Promega, Madison, WI, USA) was used to assess LDH released in the medium. The primary anti-ASC antibody (Adipogen, AG25B0006C100, 1:200) and Alexa-Fluor-594-conjugated secondary antibody (Invitrogen, Carlsbad, CA, USA, A-11037, 1:200) were used for ASC speck staining.

### 2.6. TUNEL Staining

The paraffin-embedded liver sections on the slides were dewaxed by heating at 60 °C for 2 h, followed by 3 rounds of washing in the clearing agent Xylene Substitute (Electron Microscopy Sciences, Hatfield, PA, USA, 23412-01). The samples were rehydrated by sequentially immersing the slides for 5 min in descending concentrations of ethanol (100%, 95%, 50%, 70%, and 30%). The slides were placed in preheated 0.1 M citrate buffer (pH 6) for 15 min at boiling temperature to retrieve the antigen, immersed in running tap water for 10 min, and incubated in PBS for 10 min. The sections were stained using In Situ Cell Death Detection Kit, TMR red (Roche, Indianapolis, IN, USA, 12156792910), which detects single- and double-stranded DNA breaks occurring at the early stages of apoptosis. The fluorescence signals at the damaged sites of the DNAs were obtained using an A1R-Ti2 confocal system (Nikon, Melville, NY, USA).

### 2.7. Statistics

Excel software was used to calculate statistics for the cell culture data. Differences between the two groups were compared using a two-tailed t-test. *p* < 0.05 was indicated by * and considered significant. *p* < 0.01 was indicated by **. All cell culture experiments were repeated at least 3 times on different days. Data for the animal experiments were analyzed using R version 3.6.0 (R Core Team, The R Foundation for Statistical Computing, Vienna, Austria) [32]. The Shapiro-Wilks test was first used to determine the normality of the data. The obtained small *p*-values indicated that the data were not normally distributed. Therefore, the nonparametric Mann-Whitney test was used to compare the differences between the two groups. *p* < 0.05 was indicated by * and considered significant. *p* < 0.01 was indicated by **. Power analysis showed that using 8 individual mice per group had a greater than 80% power at a significance level of 0.05, provided the probability was at least 90% that S-ELN-treated mice have lower levels of serum cytokines or expression of cytokine genes in the liver compared to control animals.

## 3. Results

### 3.1. ELNs were Extracted from a Variety of Mushrooms

**Figure 1:**
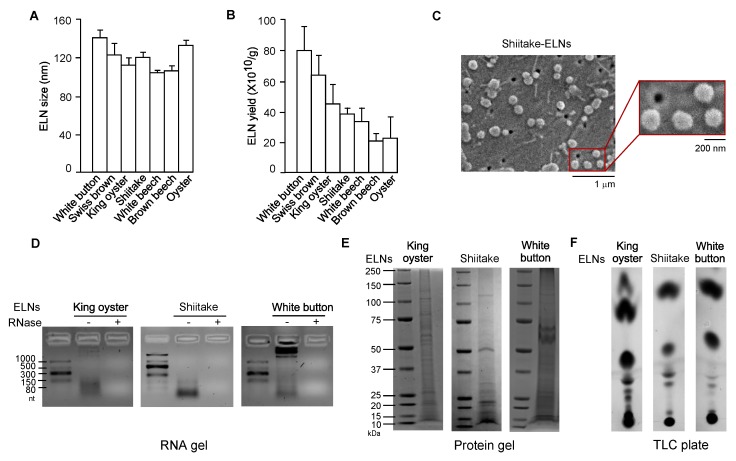
Exosome-like nanoparticles (ELNs) were isolated from a variety of mushrooms. (**A**) Sizes of ELNs from different mushrooms. (**B**) Yield of ELNs from different mushrooms. (**C**) Morphology of shiitake mushroom-derived ELNs under scanning electron microscopy (SEM). Main figure: magnification 20,000×, Inset: magnification 50,000×. (**D**) RNA gels showed the size of RNAs isolated from three mushroom-derived ELNs. The agarose gel was used. nt: Nucleotides. “−” means no RNase was incubated with RNAs and “+” means that RNase was incubated with RNAs for 30 min at 37 °C before RNAs were loaded on the gel. (**E**) Coomassie blue staining of protein gels showed various proteins in mushroom-derived ELNs. Bis-Tris protein gels were used. (**F**) Thin-layer chromatography (TLC) analysis demonstrated multiple lipid species in mushroom-derived ELNs. TLC silica gel plates were used, and lipids were visualized using CuSO_4_ phosphoric acid solution.

**Figure A1:**
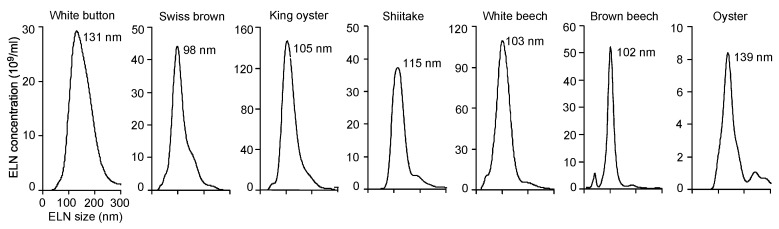
Size distribution and concentration of mushroom-derived exosome-like nanoparticles (ELNs). ELNs were extracted from 7 fresh mushrooms. ELN-containing solutions were diluted with distilled water and immediately loaded on the NanoSight NS300 instrument to measure the size and concentration of the vesicles.

Dietary ELNs have been extracted from many edible plants, including fruits, vegetables, and spices [19,21,30,31,33]. To determine whether any edible mushrooms contained ELNs, seven commonly consumed mushrooms-white button, Swiss brown, king oyster, shiitake, white beech, brown beech, and oyster-were subjected to ELN extraction procedures established in our laboratory [20]. Remarkably, ELNs were extracted from all seven mushrooms. Sizes of ELNs from different mushrooms were in the range of 100–140 nm in diameter (Figure 1A, Figure A1). Among the mushrooms tested, oyster mushroom-derived ELNs had the lowest yield of 2.3 ± 1.5 × 10^11^/g; white button mushroom-derived ELNs had the highest yield of 8.1 ± 1.6 × 10^11^/g (Figure 1B). Scanning electron microscopy (SEM) analysis showed that ELNs from shiitake mushrooms appeared as individual nanoparticles with sphere-shaped morphology (Figure 1C). Because dietary ELNs usually contain RNAs, proteins, and lipids [20,21,30], ELNs from three mushrooms (king oyster, shiitake, and white button) were subjected to biomolecule analysis. RNA analysis showed primarily small-sized RNAs from king oyster mushroom-derived ELNs and shiitake mushroom-derived ELNs (Figure 1D). Most of the RNAs from white button mushroom-derived ELNs were large-sized RNAs, although a small portion was small in size (Figure 1D). Proteins of ELNs from these three mushrooms ranged widely in mass from 10 kDa to 150 kDa (Figure 1E). TLC analysis demonstrated that ELNs from these three mushrooms contained a series of lipids (Figure 1F). Therefore, nanoparticle-scaled ELNs were extracted from mushrooms and contained RNAs, proteins, and lipids.

### 3.2. S-ELNs Inhibited NLRP3 Inflammasome Activation and IL-6 Release

**Figure A2:**
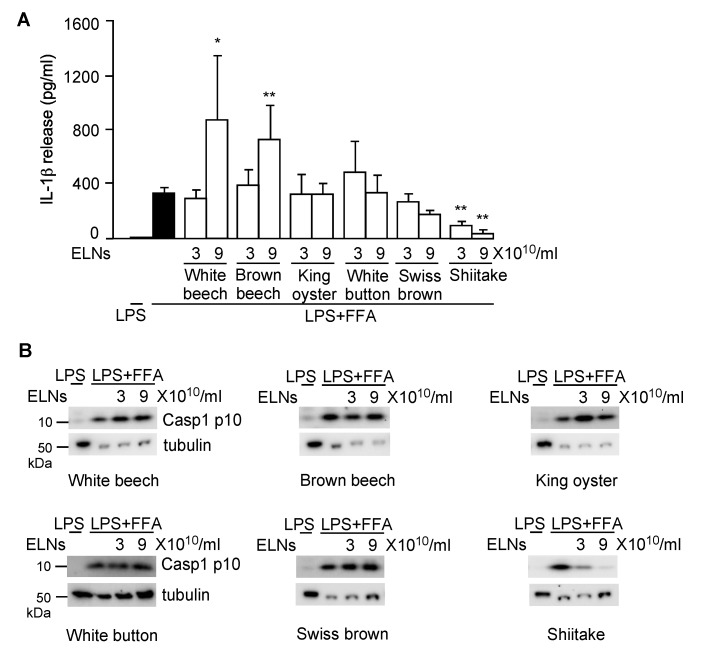
Exosome-like nanoparticles (ELNs) from shiitake mushrooms, but not other mushrooms, inhibited nucleotide-binding domain and leucine-rich repeat related (NLR) family, pyrin domain containing 3 (NLRP3) inflammasome activity. Effects of ELNs from 6 mushrooms on interleukin (IL)-1β secretion (**A**) and Caspase 1 (Casp1) autocleavage (**B**) when the NLRP3 inflammasome was activated. Primary macrophages were pre-incubated with ELNs from 6 mushrooms, including white beech, brown beech, king oyster, white button, Swiss brown, and shiitake, for 16 h. Afterward, cells were incubated with lipopolysaccharide (LPS) and free fatty acid (FFA) to activate the NLRP3 inflammasome. Tubulin is a loading control in the immunoblot analysis. Cells treated with LPS alone served as a negative control for NLRP3 inflammasome activation. * *p* < 0.05, ** *p* < 0.01 relative to cells treated with LPS+FFA (black bar).

Next, to assess whether any mushroom-derived ELNs suppressed NLRP3 inflammasome activity, ELNs from six mushrooms—white beech, brown beech, king oyster, white button, Swiss brown, and shiitake—were preincubated with the primary macrophages, BMDMs, followed by activation of the NLRP3 inflammasome using LPS and FFA sodium palmitate. NLRP3 inflammasome activity was assessed by two downstream products of the inflammasome activation: IL-1β release in the culture medium and the level of Casp1 autocleavage product Casp1 p10 in cell lysates [34,35]. ELNs from white beech and brown beech mushrooms at a low concentration (3 × 10^10^/mL) had no effects on IL-1β release but began to promote IL-1β secretion when their concentration reached 9 × 10^10^/mL (Figure A2A). ELNs from king oyster, white button, and Swiss brown mushrooms did not affect IL-1β secretion (Figure A2A). ELNs from these five mushrooms had no significant impacts on the Casp1 p10 level (Figure A2B). In contrast, ELNs from shiitake mushrooms remarkably inhibited both IL-1β secretion and Casp1 p10 levels (Figure A2A,B).

**Figure 2:**
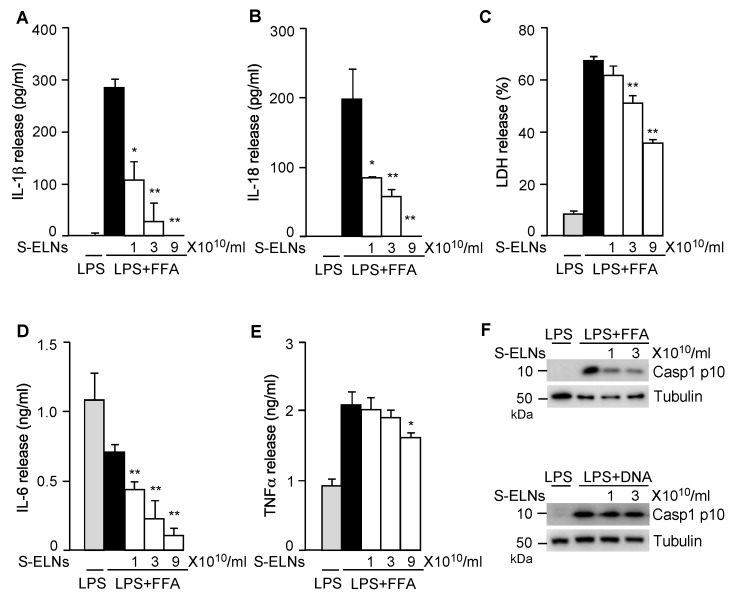
Shiitake mushroom-derived exosome-like nanoparticles (S-ELNs) suppressed nucleotide-binding domain and leucine-rich repeat related (NLR) family, pyrin domain containing 3 (NLRP3) inflammasome activity and interleukin (IL)-6 release. Bone marrow-derived macrophages (BMDMs) were preincubated with S-ELNs for 16 h, then treated with lipopolysaccharide (LPS) and free fatty acid (FFA) to activate the NLRP3 inflammasome. The cell-free culture medium was used to assess the levels of cytokines, including IL-1β (**A**), IL-18 (**B**), IL-6 (**D**), and tumor necrosis factor α (TNFα) (**E**), and lactate dehydrogenase (LDH) release (**C**). * *p* < 0.05, ** *p* < 0.01 relative to cells treated with LPS+FFA (black bar). The cell lysate was subjected to immunoblot analysis to measure the level of Caspase 1 (Casp1) p10 (Casp1 autocleavage product) when the NLRP3 and Absent in Melanoma 2 (AIM2) inflammasome were activated by LPS+FFA and LPS+DNA, respectively. (**F**). Tubulin is a loading control.

Activation of the NLRP3 inflammasome triggers other downstream pathways, including pyroptotic cell death and IL-18 secretion [11,12]. S-ELN treatment dose-dependently inhibited the release of both IL-1β (Figure 2A) and IL-18 (Figure 2B). LDH release assay, which was used to assess pyroptosis upon NLRP3 inflammasome activation [14,28], showed that S-ELNs dose-dependently suppressed pyroptosis (Figure 2C). Treatment of the priming signal LPS with macrophages induced secretion of two other potent pro-inflammatory cytokines, IL-6, and TNFα, through the Nuclear Factor kappa-light-chain-enhancer of activated B cells (NF-ĸB) pathway [36]. Interestingly, S-ELNs strongly suppressed IL-6 secretion (Figure 2D) but had very mild inhibitory effects on TNFα secretion (Figure 2E), indicating that S-ELNs may contain active agents that specifically inhibit the secretion of IL-6.

**Figure A3:**
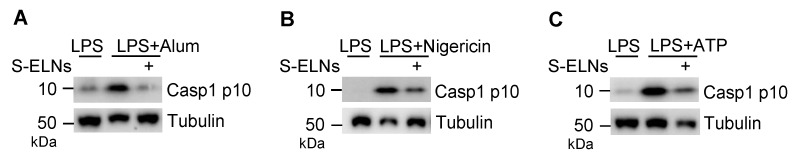
Shiitake mushroom-derived exosome-like nanoparticles (S-ELNs) suppressed the nucleotide-binding domain and leucine-rich repeat related (NLR) family, pyrin domain containing 3 (NLRP3) inflammasome activated with three other stimuli. Bone marrow-derived macrophages (BMDMs) were treated with S-ELNs (3 × 10^10^/mL) for 16 h. Afterward, the macrophages were primed with lipopolysaccharide (LPS) for 3 h, incubated with alum (**A**), nigericin (**B**), or ATP (**C**) to activate the NLRP3 inflammasome. Cell lysates were used for immunoblot analysis. The level of Caspase 1 (Casp1) p10 indicated Casp1 autocleavage during inflammasome activation. Tubulin was a loading control.

The NLRP3 inflammasome is activated by various pathogen-derived agents or endogenous stress molecules [11,12,13]. When three other activators of the NLRP3 inflammasome-alum, nigericin, and ATP-were used, S-ELNs suppressed the NLRP3 inflammasome activated by these stimuli (Figure A3). Another related inflammasome—the AIM2 inflammasome, composed of AIM2, ASC, and Casp1 subunits—was activated by cytosolic DNA during bacterial and viral infection [37,38,39]. When the AIM2 inflammasome is activated, its subunit Casp1 also autocleaves itself to generate Casp1 p10. Although S-ELNs strongly inhibited the level of Casp1 p10 during NLRP3 inflammasome activation, they had no impact on the Casp1 p10 level when the AIM2 inflammasome was activated (Figure 2F), suggesting that S-ELN treatment specifically inhibited NLRP3 inflammasome activity and did not compromise the general functions of macrophages.

### 3.3. S-ELNs Inhibited Assembly of the NLRP3 Inflammasome and Decreased both Protein and mRNA Levels of the Il1b Gene

**Figure 3:**
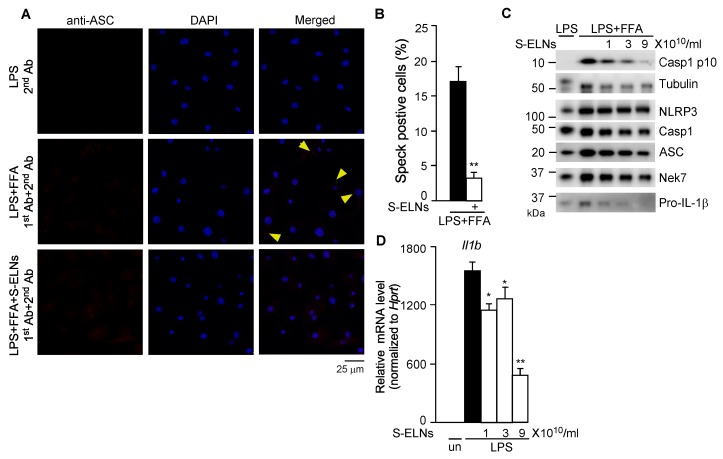
Shiitake mushroom-derived exosome-like nanoparticles (S-ELNs) prevented inflammasome formation and inhibited both protein and mRNA levels of the *Il1b* gene. (**A**) Representative pictures of immunofluorescence staining in macrophages. Magnification: 60×. Cells were treated with S-ELNs (3 × 10^10^/mL) for 16 h. Afterward, Caspase 1 (Casp1) inhibitor VX765 (10 µM) was added to the cells for 30 min, followed by lipopolysaccharide (LPS) and free fatty acid (FFA) treatment. Macrophages were subjected to immunofluorescence staining using a primary antibody (1st Ab) and secondary antibody (2nd Ab). The nucleotide-binding domain and leucine-rich repeat related (NLR) family, pyrin domain containing 3 (NLRP3) inflammasome was visualized as red specks (indicated by yellow arrows). 4′,6-Diamidino-2-phenylindole (DAPI) was used to stain nuclei. (**B**) The percentage of speck positive cells was decreased by the pre-treatment of S-ELNs. ** *p* < 0.01 relative to cells treated with LPS+FFA (black bar). (**C**) Protein levels of NLPR3, apoptotic speck protein containing a caspase recruitment domain (ASC), Casp1, and never in mitosis gene a (NIMA)-related expressed kinase 7 (Nek7) were not significantly altered by S-ELN treatment. (**D**) S-ELNs decreased the expression of the *Il1b* gene. * *p* < 0.05, ** *p* < 0.01 relative to cells primed with LPS alone (black bar). un: un-treated naïve macrophages.

Upon NLRP3 inflammasome activation, NLRP3 recruits ASC and Casp1 to assemble a multiprotein complex with a high molecular mass, which can be detected as a speck under confocal microscopy [35,40]. After BMDMs were treated with LPS and FFA, cells were subjected to immunofluorescence staining using an anti-ASC antibody. ASC specks were detected in many macrophages, whereas pre-treatment of cells with S-ELNs significantly reduced the formation of ASC specks (Figure 3A,B), indicating that S-ELNs inhibited the assembly of the inflammasome complex. NLRP3, ASC, and Casp1 subunits were not significantly affected by S-ELNs (Figure 3C). Recently, never in mitosis gene a (NIMA)-related expressed kinase 7 (Nek7) was identified as a key mediator of the NLRP3 inflammasome [40,41,42]. However, the protein level of Nek7 was not altered by S-ELNs (Figure 3C). Therefore, S-ELNs may target upstream events of the NLRP3 inflammasome to block inflammasome assembly.

Interestingly, S-ELNs remarkably inhibited the protein level of Pro-IL-1β (Figure 3C). Pro-IL-1β protein is dramatically increased in macrophages upon LPS treatment because LPS enhances the expression of the *Il1b* gene [43]. Therefore, the effects of S-ELNs on the expression of the *Il1b* gene were assessed, and S-ELNs were found to dose-dependently suppress the expression of the *Il1b* gene triggered by LPS treatment (Figure 3D).

### 3.4. S-ELNs Protected Mice from GalN/LPS-Induced Acute Liver Injury

**Figure 4:**
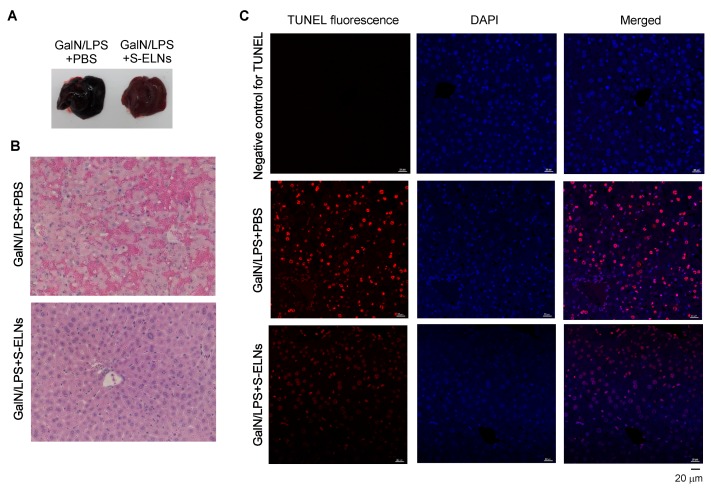
Shiitake mushroom-derived exosome-like nanoparticles (S-ELNs) protected animals from D-galactosamine (GalN)/lipopolysaccharide (LPS)-triggered liver damage. C57BL/6J mice were administered with solvent phosphate-buffered saline (PBS) or S-ELNs in PBS using intraperitoneal injection. 48 h later, the mice received a GalN/LPS mixture through intraperitoneal injection. The mice were sacrificed after 6 h, and their serum and liver were collected for analysis. (**A**) Representative pictures of whole livers of mice. (**B**) Representative images of Haemotoxylin and Eosin (H&E) staining of liver tissues. Magnification: 40×. (**C**) Representative images of terminal deoxynucleotidyl transferase dUTP nick end labeling (TUNEL) staining of liver tissues. Magnification: 40×. In the negative control for TUNEL staining, the sections were incubated with TUNEL mixture lacking the enzyme terminal deoxynucleotidyl transferase (TdT), which adds fluorescence-labeled dUTP at the single-and double-stranded DNA breaks. DAPI: 4′,6-Diamidino-2-phenylindole. *N* = 6−8/group.

To assess the functional significance of S-ELNs in the disease model of FHF, S-ELNs were injected intraperitoneally into mice. 48 h later, a mixture of GalN and LPS was administered through intraperitoneal injection to induce acute liver injury. Mice were sacrificed after 6 h for analysis. Consistent with the literature [6,44], the livers from mice challenged with the GalN/LPS mixture appeared black and sick (Figure 4A). Interestingly, livers from mice pre-treated with S-ELNs showed a much healthier appearance (Figure 4A). Histologically, livers from mice injected with GalN/LPS showed extensive hemorrhage and cell death in the Haemotoxylin and Eosin (H&E)-stained sections, whereas pre-treatment with S-ELNs alleviated these pathological alterations (Figure 4B). TUNEL staining of liver sections confirmed that there were much fewer apoptotic cells in the livers of GalN/LPS-S-ELN-treated mice compared to the mice challenged with GalN/LPS (Figure 4C).

**Figure 5:**
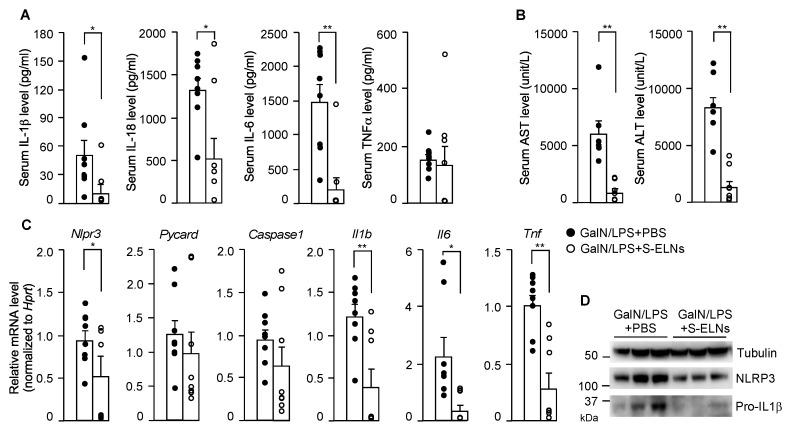
Shiitake mushroom-derived exosome-like nanoparticles (S-ELNs) reduced inflammation and improved liver function in D-galactosamine (GalN)/lipopolysaccharide (LPS)-triggered acute liver damage. The serum and liver samples from the same animals in Figure 4 were subjected to further analysis. (**A**) Levels of cytokines, including interleukin (IL)-1β, IL-18, IL-6, and tumor necrosis factor α (TNFα) in the serum. (**B**) Levels of aspartate aminotransferase (AST) and alanine aminotransferase (ALT) in the serum. (**C**) mRNA analysis of liver tissues. (**D**) Immunoblot analysis of liver samples. Each dot in the bar graphs represents one mouse in each group. N = 6–8/group. * *p* < 0.05, ** *p* < 0.01 relative to mice challenged with GalN/LPS+phosphate-buffered saline (PBS) (bar with black dots).

**Figure A4:**
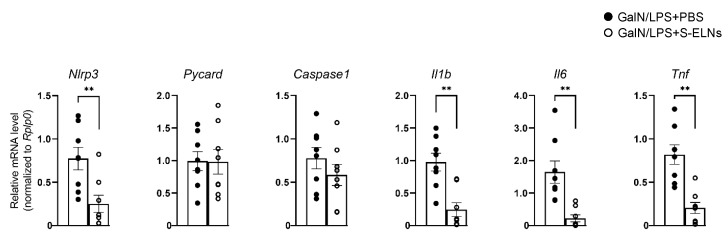
Treatment of shiitake mushroom-derived exosome-like nanoparticles (S-ELNs) reduced the expression of inflammatory genes. The mRNAs of livers from the same animals in Figure 5 were used to run a quantitative PCR (qPCR) analysis of the housekeeping gene *Rplp0*. The relative mRNA levels of inflammatory genes were normalized to the *Rplp0* gene. Each dot in the bar graphs represents one mouse in each group. *N* = 8/group. * *p* < 0.05, ** *p* < 0.01 relative to mice challenged with D-galactosamine (GalN)/lipopolysaccharide (LPS)+ phosphate-buffered saline (PBS) (bar with black dots).

In the serum, the levels of two downstream cytokines of NLRP3 inflammasome activation, IL-1β, and IL-18, decreased significantly after S-ELN treatment (Figure 5A). In addition, S-ELNs remarkably reduced the IL-6 serum level but had no impact on the TNFα serum level (Figure 5A). The elevated levels of serum ALT and AST after GalN/LPS injection were mitigated after S-ELN treatment (Figure 5B). At the molecular level, S-ELNs reduced expression of the *Nlrp3, Il1b*, *Il6*, and *Tnf* genes in the liver, but did not affect the mRNA level of the *Pycard* (*Asc*) and *Caspase1* genes, when these genes were normalized with the reference gene *Hprt* (Figure 5C). This finding remained the same when the expression of these genes was normalized to another housekeeping gene *Rplp0* (Figure A4). At the protein level, S-ELN treatment accordingly decreased Pro-IL-1β and NLRP3 in the GalN/LPS-challenged livers (Figure 5D). Overall, the physiological and molecular evidence suggested that S-ELN pre-treatment alleviated the severity of GalN/LPS-induced acute liver injury in mice.

## 4. Discussion

Recently, dietary ELNs have been extracted from fruits (such as grapes [21], grapefruit [17,19], apples [33], and coconut water [45]); vegetables (such as carrots [21] and broccoli [31]); and spices (such as ginger [19,20,30] and turmeric [20]). However, it was not known whether edible mushrooms contained any ELNs. Our research, for the first time in the literature, has demonstrated that ELNs can be extracted from edible mushrooms. Sizes of ELNs from seven edible mushrooms ranged from 100 nm to 140 nm in diameters (Figure 1A). Their sizes were comparable to those of dietary ELNs extracted from edible plants [21,33,45]. Mushroom-derived ELNs contained RNAs, proteins, and lipids (Figure 1D–F) and, therefore, were similar in composition to other dietary ELNs [20,21,30].

Shiitake mushrooms have been widely used as a food and medicine for centuries in China, Japan, and Korea and have become a popular healthy food in Europe and North America [46,47]. Extracts of shiitake mushrooms have shown strong anti-inflammatory functions in the murine macrophage cell line RAW 264.7 [48]. The polysaccharide lentinan from shiitake mushrooms has strong anti-tumor activities and also inhibits bacterial, viral, and parasitic infections [49,50]. The small biomolecule eritadenine in shiitake mushrooms has the ability to lower cholesterol in rats [51]. Here, we found that ELNs from shiitake mushrooms strongly inhibited NLRP3 inflammasome activity but not AIM2 inflammasome activity (Figure 2). This function apparently is not mediated by the well-characterized biomolecule lentinan in shiitake mushrooms because lentinan was shown to specifically inhibit activation of the AIM2 inflammasome, but not the NLRP3 inflammasome [52]. Therefore, our research indicated that the newly identified component ELNs in shiitake mushrooms contribute to their anti-inflammatory functions.

FHF is a life-threatening health condition with poor prognosis [2,3]. The high mortality of this condition underscores the urgency of identifying new therapeutic interventions. Suppressing NLRP3 inflammasome activity has been shown to alleviate the severity of GalN/LPS-triggered acute liver injury in mice, which mimics many characteristics of FHF in clinical settings [6,8]. S-ELNs demonstrated potent anti-NLRP3 inflammasome activity in primary macrophages, but, more importantly, when S-ELNs were administered to mice through intraperitoneal injection, they protected mice from GalN/LPS-triggered acute liver damage (Figure 4 and Figure 5). Our proof-of-principal studies suggested that ELNs from shiitake mushrooms represent a new potential intervention strategy to curb FHF. The membrane of exosomes or ELNs encloses the biomolecules in the vesicles and, therefore, protects these biomolecules from degradation [53,54,55]. In addition, the abundance of ELNs in shiitake mushrooms (Figure 1B) would facilitate the generation of large quantities of nanoparticles for translational applications.

In the future, further studies are needed to define the exact bioactive agents in S-ELNs that are responsible for their anti-NLRP3 inflammasome functions. In addition, S-ELNs decreased both protein and mRNA levels of the *Il6* and *Il1b* genes in GalN/LPS-induced liver damage, indicating that other anti-inflammatory functions of S-ELNs may contribute to ameliorating the severity of this disease model. Further investigation is necessary to delineate how S-ELNs suppress the expression of these potent cytokine genes.

## 5. Conclusions

In summary, we found that common edible mushrooms contain ELNs composed of lipids, RNAs, and proteins. Among mushroom-derived ELNs, those from shiitake mushrooms, S-ELNs, demonstrated strong anti-inflammatory activity. S-ELNs blocked the formation of the NLRP3 inflammasome and inhibited downstream events of inflammasome activation, including cytokine secretion, Casp1 autocleavage, and pyroptotic cell death. More importantly, pre-treatment with S-ELNs protected mice from GalN/LPS-triggered acute liver damage; therefore, S-ELNs represent a promising new bioactive agent with the potential for the treatment of FHF.
